# Stromal Cells Underlining the Paths From Autoimmunity, Inflammation to Cancer With Roles Beyond Structural and Nutritional Support

**DOI:** 10.3389/fcell.2021.658984

**Published:** 2021-05-25

**Authors:** Amanda M. Honan, Zhibin Chen

**Affiliations:** ^1^Department of Microbiology and Immunology, Miller School of Medicine, University of Miami, Miami, FL, United States; ^2^Sylvester Comprehensive Cancer Center, Miller School of Medicine, University of Miami, Miami, FL, United States

**Keywords:** stromal cells, secondary lymphoid organs, autoimmunity, cancer, non-lymphoid tissues

## Abstract

Stromal cells provide structural support and nutrients in secondary lymphoid organs and non-lymphoid tissues. However, accumulating evidence suggests that a complex relationship exists between stromal cells and immune cells. Interactions between immune cells and stromal cells have been shown to influence the pathology of both autoimmunity and cancer. This review examines the heterogeneity of stromal cells within the lymph node and non-lymphoid tissues during both homeostatic and inflammatory conditions, in particular autoimmunity and cancer, with the goal of better understanding the complex and apparently paradoxical relationship between these two classes of diseases. The review surveys potential novel mechanisms involving the interactions between stromal cells and immune cells which may contribute to the development, pathology and underlying connection between autoimmunity and cancer, including potential pathways from autoimmune inflammation to either “hot” or “cold” tumors. These interactions may provide some insights to explain the rising incidence of both autoimmunity and cancer in young women in industrialized countries and have the potential to be exploited in the development of new interventions for preventions and treatments of both autoimmune diseases and cancer.

## Introduction

Stromal cells are commonly known for their role in providing support and nutrients in both the secondary lymphoid organs and non-lymphoid tissues. However, recent studies have illustrated the expansive role of stromal cells along with the characterization of the diverse group of cells that constitute the stromal cell compartment. The stromal cell compartment is comprised of many types of cells including but not limited to endothelial cells, mesenchymal stromal cells, and pericytes. Within these larger categories are even more subsets of cells which help shape both the stromal compartment and organ microenvironments (Chang and Turley, 2015; Jalkanen and Salmi, 2020; Krishnamurty and Turley, 2020). This heterogeneity is conserved from the healthy setting to the inflammatory setting such as in autoimmune diseases and cancer (Petrova and Koh, 2018; Koliaraki et al., 2020). Understanding these extensively diverse cell types and their functions and interactions with immune cells in both the healthy setting and the inflammatory setting may help better understand the influence of stromal cells in disease pathology, leading to new approaches for the treatment and prevention of inflammatory diseases including autoimmune disorders and cancer, two of the major disease burdens on human beings.

On the “surface” autoimmunity and cancer appear to be at the opposite end of the spectrum of immune-related diseases. Autoimmune diseases are caused by the immune system wreaking havoc on the body’s healthy tissues, while cancer represents a failure of the immune system to destroy malignant cells. The recent breakthrough in cancer immune therapy coincidently brought a broader attention to at least one aspect of the intricacy in the relationship between autoimmunity and cancer — autoimmune damage is not just off-target toxicity but also an indicator of effective anti-tumor immunity (Attia et al., 2005; Postow et al., 2018). More perplexingly, there is a long-standing suspicion that autoimmunity might increase the risk of cancer development (Volkers, 1999). That link has been largely regarded with a relevance only to rare cases of cancer, until the recent epidemiological findings of alarming trends of rising cancer incidences in young adults (Anderson et al., 2018; Blaser and Chen, 2018; Kehm et al., 2019; Miller et al., 2020), with the demographic and other evidence from the stomach cancer study suggesting autoimmunity as a potential etiological factor in cancer development at the population level (Anderson et al., 2018; Blaser and Chen, 2018; Kehm et al., 2019; Miller et al., 2020). Whether and how autoimmunity may contribute the rising incidences of early onset cancer in humans remains to be examined. On the other hand, studies using independent approaches with distinct animal models have provided strong evidence for the causality of autoimmune T cells in cancer development (Nguyen et al., 2013; Miska et al., 2018).

Nevertheless, it remains largely unclear how exactly T cells in inflammatory settings may promote cancer development. In this review, we explore that question from the angle of the stromal cell biology with a focus on stromal–immune interactions, keeping in mind that the extensive changes in the stromal compartment, such as tissue remodeling and fibrosis, are often associated with both autoimmune disorders and cancer.

## Heterogeneity of Stromal Cells in Healthy Tissues

Stromal cells are essential components in both lymphoid and non-lymphoid tissues. In central lymphoid organs such as the thymus and bone marrow, it has been well-recognized that they play an essential role in supporting lymphocyte development and selection (Petrie and Zuniga-Pflucker, 2007; Mercier et al., 2011). In the periphery, a complex array of stromal cell subsets have been well- characterized. With the constrain of the scope and limitation of our knowledge basis, we will focus this review on the more recent studies on stromal cell and T lymphocyte interaction in the periphery including lymph nodes and non-lymphoid tissues.

**FIGURE 1:**
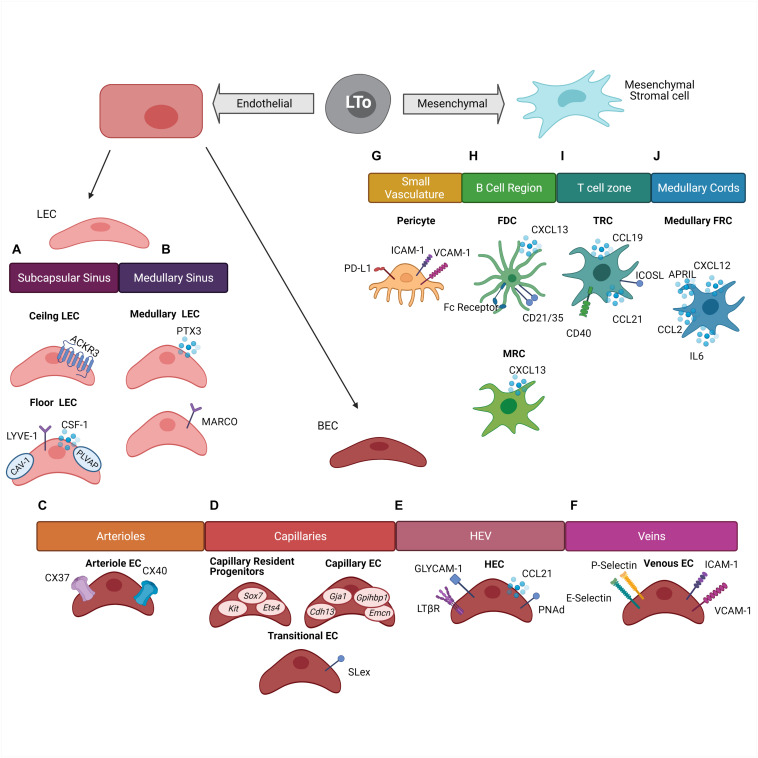
Stromal cell heterogeneity within the lymph node. Stromal cells within the lymph node are derived from two cell lineages which originated from the Lymphoid Tissue organizer (LTo) cells: endothelial lineage and mesenchymal lineage. **(A)** Subcapsular sinus lymphatic endothelial cells (LEC) are divided into two groups. Ceiling LEC expressing Atypical Chemokine Receptor 3 (ACKR3) and floor LEC expressing Colony Stimulating Factor 1 (CSF-1), Lymphatic Vessel Endothelial Receptor 1 (LYVE-1), Caveolin-1 (CAV-1), and Plasmalemma Vesicle Associated Protein (PLVAP). **(B)** Medullary sinus LEC are divided into two groups based off of Pentraxin (PTX3) or Macrophage Receptor With Collagenous Structure (MARCO) expression. **(C)** Arterial endothelial cells (EC) express Connexin 37 (CX37) and Connexin 40 (CX40). **(D)** Capillary BEC are divided into three groups. Capillary resident progenitors express *Kit*, *Sox7*, and *Ets4*, Capillary EC express Cdh13, Gja1, Gpihbp1, and Emcn, and Transitional EC express Sialyl Lewis^*X*^ (SLex). **(E)** High endothelial venules (HEV) are composed of high endothelial cells (HEC) and express Glycosylation-Dependent Cell Adhesion Molecule-1 (GLYCAM-1), Lymphotoxin Beta Receptor (LTβR), Chemokine Ligand 21 (CCL21), and Peripheral Lymph Node Addressin (PNAd). **(F)** Veins are composed of Venous EC which express E-selectins, P-selectins, Intercellular Adhesion Molecule 1 (ICAM-1), and Vascular Cell Adhesion Molecule 1 (VCAM-1). **(G)** Small vasculature is surrounded by pericytes which express Programmed Death-Ligand 1 (PD-L1), ICAM-1, and VCAM-1. **(H)** The B cell region is comprised of Follicular Dendritic Cells (FDC) which express Fc-Receptor, CD21/CD35 complex, and CXCL13. Marginal reticular cells express CXCL13. **(I)** The T cell zone is composed of T cell zone reticular cells expressing CD40, CCL21, and CCL19, and Inducible T Cell Costimulatory Ligand (ICOSL). **(J)** The medullar cords are comprised of Medullary Fibroblast Reticular Cells (FRC) which express CXCL12, CCL2, IL-6, and A Proliferation-Inducing Ligand (APRIL).

Within the lymph node, stromal cells are derived from either mesenchymal or endothelial origins (Figure 1). These two cell origins can differentiate further into many cell types which include but are not limited to lymphatic endothelial cells (LEC), blood endothelial cells (BEC), fibroblasts, and pericytes. Within these subsets there are even more diversity, as each subset is comprised of a heterogeneous group of cells which can vary in location and function throughout the lymph node (Chang and Turley, 2015; Krishnamurty and Turley, 2020). Stromal cell populations can also be found in non-lymphoid tissues throughout the body. Non-lymphoid tissue stromal cells are also derived from the endothelial and mesenchymal lineages and function similarly to lymph node stromal cells (Koliaraki et al., 2020; Krausgruber et al., 2020). In this section, we will explore the heterogeneity of stromal cells within the lymph node and the non-lymphoid tissues under homeostatic conditions (Table 1).

**TABLE 1:** Heterogeneity in function of lymph node and non-lymphoid tissue stromal cells.

Cell Type	Lineage	Site	Function							
			*Fluid drainage*	*Lymphocyte trafficking*	*Nutrient delivery*	*Antigen presentation*	*Tolerance induction*	*Immune suppression*	*Innate immune response*	*Angio-genesis*
*Lymphatic Endothelial Cell*	Endothelial	Line lymphatic vessels	X	X	X	X	X	X	X	
*Blood Endothelial Cell*	Endothelial	Line blood vessels	X	X	X				X	X
*Fibroblast*	Mesenchymal	B cell region, T cell zone, medullary cord, various non-lymphoid tissue		X		X	X	X	X	
*Pericyte*	Mesenchymal	Surrounding small blood vessels		X				X	X	X

*Stromal cells can be found in both the lymph node and various non-lymphoid tissues throughout the body helping to maintain the proper structure and microenvironment within these tissues, as well as interact with various types of immune cells. These populations of stromal cells are extremely diverse with many new functions recently being discovered. This table outlines the heterogeneity of four main subsets of stromal cells: lymphatic endothelial cells, blood endothelial cells, fibroblasts, and pericytes. Additionally, various functions of stromal cells within the lymph node and non-lymphoid tissue are also outlined. These functions maybe specific to the geographically location of the stromal cell subset.*

### Endothelial Cell Populations in the Lymph Node

Lymphatic endothelial cell can be found lining both the afferent and efferent lymphatic vessels. Traditionally, it was believed that their main role was to drain lymph from surrounding tissues in and out of the lymph node, but more recently exciting new functions in antigen presentation and immune tolerance induction have been discovered along with the identification of multiple new subsets of LEC (Jalkanen and Salmi, 2020).

Recent studies in both mice and humans have found transcriptionally distinct populations of LEC residing in distinct geographic location within the lymph node (Iftakhar et al., 2016; Takeda et al., 2019; Fujimoto et al., 2020; Xiang et al., 2020). The lymph node has a series of sinuses which lymph from the afferent lymphatic vessels drains. These sinuses then empty into the efferent lymphatic vessels which carry lymph out of the draining lymph node and to the next lymph node in the chain. These vessels and sinuses are lined with a continuous layer of LEC. Additionally, sites where the lymphatic vessels open into the sinuses contain LEC lined valves (Jalkanen and Salmi, 2020). Within the various vessels, sinuses, and valves are distinct populations of LEC. A study by Iftakhar et al. (2016) found that 400 genes were upregulated and almost 300 genes were downregulated more than eight-fold in pooled cortical and medullary sinus LEC compared to subcapsular LEC. The differentially expressed genes belonged to several different categories including metabolic functions, stress response, and immune response (Iftakhar et al., 2016), indicating the possibility of niche-specific functions of LEC even within the same lymph node. Heterogeneity also exists within the LEC populations in the subcapsular and medullary sinus. Within the subcapsular sinus, ceiling LEC express caveolin-1 (CAV-1) and plasmalemma vesicle-associated protein (PLVAP) which create size exclusion diaphragms and regulate the filtering of soluble antigens into the conduits and the migration of lymphocytes through the LEC floor (Rantakari et al., 2015; Iftakhar et al., 2016; Jalkanen and Salmi, 2020); CSF-1 secretion by floor LEC help maintain the subcapsular macrophage niche (Mondor et al., 2019; Pikor et al., 2021). Ceiling LEC express the atypical chemokine receptor ACKR3 allowing for the scavenging of CCL21 (Ulvmar et al., 2014; Pikor et al., 2021) (Figure 1A). The LEC population in the medullary sinus can be divided into two groups based on the differential expression of PTX3 and MARCO (Xiang et al., 2020; Pikor et al., 2021) (Figure 1B). It is also now well-recognized that the various LEC subsets have a critical role in antigen presentation (Cohen et al., 2010; Norder et al., 2012; Dubrot et al., 2014; Berendam et al., 2019; Jalkanen and Salmi, 2020). LEC express both the MHC class I (MHCI) and MHC class II (MHCII) molecule. MHCII can be endogenously expressed as well as acquired from dendritic cells through transcytosis (Dubrot et al., 2014). LEC also express molecules such as the transporter associated with antigen processing and the invariant chain indicating the ability for LEC to process and present cytosolic as well as exogenous antigen (Santambrogio et al., 2019).

Lymphatic endothelial cells can serve a critical role in immune tolerance induction through presentation of self-antigens as a potential mechanism of immune regulation. Medullary and cortical LEC can transcribe selected peripheral antigens in an autoimmune regulator (AIRE) independent manner. Peripheral antigens presented by the LEC subset include melanocyte-specific tyrosinase, intestinal epithelial protein A33, and pancreatic prohormone (PYY) (Cohen et al., 2010). LEC also express the inhibitory molecule programmed cell death ligand 1 (PD-L1) (Tewalt et al., 2012). PD-L1 expression by LEC has been linked to the potential elimination of autoreactive CD8^+^ T cells (Tewalt et al., 2012). Additionally, LEC play an important role in the migration of T_reg_ cells into the lymph node from afferent lymphatic vessels, an important mechanism for establishing tolerance. This migration is dependent on LTβR expression by LEC which interact with LTαβ expressed by T_reg_ cells. LTβR interaction was shown to be not needed for T_reg_ migration from the blood through high endothelial venules (HEV), which also express LTβR, or required by other T cell populations. These results illustrated a novel mechanism for T_reg_ migration through interactions with LEC (Brinkman et al., 2016).

The other major endothelial cell lineage in lymph nodes is BEC. BEC are comprised of two main groups. These groups include the endothelial cells within the HEV and the non-HEV cells. Within the lymph node BEC expand and create a capillary network. This network is responsible for the delivery of solutes, metabolites, and oxygen to the lymphocytes along with control of fluid between tissues. In the lymph node this network of vessels ends at the HEV (Krishnamurty and Turley, 2020), wherein a group of specialized BEC express a family of adhesion molecules and chemokines in order to capture lymphocytes and facilitate their entry into the lymph node. Maintenance of HEV function is necessary for proper trafficking and regulation of lymphocytes (Krishnamurty and Turley, 2020).

Like LEC, BEC are comprised of diverse subsets in terms of both phenotype and function. Brulois et al. (2020) examined sorted BEC populations from the peripheral lymph nodes of mice. Within the BEC population eight transcriptionally distinct subsets were discovered. These subsets included an arteriole endothelial cell population, two venous endothelial cell populations, a high endothelial cell population, a non-high endothelial venous cell population, and five capillary endothelial cell phenotypes (Brulois et al., 2020).

Arteriole endothelial cells express *Gja5* and *Gja4* which encode connexin 37 (CX37) and connexin 40 (CX40) (Figure 1C). Capillary endothelial cells were identified through the expression of *Gja1*, *Cdh13*, *Gpihbp1*, and *Emcn*. This gene profile has been previously shown to identify capillary endothelial cells (Lee et al., 2014) (Figure 1D). Within the capillary endothelial cell populations, a transitional capillary phenotype and a resident regenerative population have been identified. The capillary transitional phenotype presented a transcriptional profile as well as a location which was intermediate between the other capillary endothelial cell populations and the high endothelial cells (HEC). This population expressed glycotopes, including 6-sulfo-SLex, which allow for lymphocyte tethering under flow, indicating the ability for lymphocytes to interact with the endothelium prior to entering the HEV (Brulois et al., 2020) (Figure 1D). The resident regenerative capillary population presented features that are commonly exhibited by multipotent progenitor cells. This population expressed genes commonly upregulated in stem cells and progenitor cells such as *Sox7*, *Kit*, and *Ets4* (Figure 1D). It is believed this population may play a role in basal endothelial proliferation and vascular neogenesis (Brulois et al., 2020). HEC express high levels of peripheral node addressin (PNAd) and CCL21. This expression mediates the entry of naïve lymphocytes into the lymph node (Mionnet et al., 2011). HEC also express LTβR and glycosylation-dependent cell adhesion molecule 1 (GLYCAM1) (Figure 1E) (Onder et al., 2013; Veerman et al., 2019). Furthermore, medullary venous endothelial cells are characterized by the expression of both E- and P-selectins along with ICAM-1 and VCAM-1 (Figure 1F). It is believed this population may preferentially mediate myeloid cell associated inflammation (Brulois et al., 2020).

### Mesenchymal-Derived Cell Populations in the Lymph Node

During lymph node development mesenchymal cells divide the lymph node into the B and T cell regions (Koliaraki et al., 2020). Mesenchymal cells in the B cell domain are characterized as expressing the B cell attracting chemokine CXCL13 and can further differentiate into marginal reticular cells (MRC) and follicular dendritic cells (FDC). MRC help to support the delivery of antigens to B cells and shape the microenvironment, while FDC specialize in the retention, storage, and presentation of unprocessed antigens to B cells (Heesters et al., 2014; Rodda et al., 2018; Denton et al., 2019) (Figure 1H). Recently, two distinct FDC populations were identified using genetic targeting of the *Cxcl13* expressing cells and single cell transcriptomics. The light zone (LZ)-FDC population represented the classical FDC profile expressing both complement and Fc-receptors as well as genes associated with intracellular stiffness. The LZ-FDC gene profile supported the mechanical properties needed to present antigen. The dark zone (DZ)-FDC were shown to express lower levels of these receptors and tended to express a somewhat hybrid phenotype between the LZ-FDC and other B cell region reticular cell (Pikor et al., 2020).

The mesenchymal-derived cells in the T cell zone are most commonly referred to as the T cell zone reticular cells (TRC). This population can be identified by the expression of CCL19 and CCL21 (Rodda et al., 2018). The TRC population forms a meshwork within the T cell zone attracting both T cells and dendritic cells and facilitating their interaction. Additionally, TRC can play a role in both inducing an immune response and inducing tolerance (Koliaraki et al., 2020). TRC can express costimulatory molecules such as ICOSL and CD40, or cytokine IL-6 which helps to promote the production of IL-2 and TNF by CD8^+^ T cells (Brown et al., 2019) (Figure 1I). TRC can also respond to pathogen associated molecular pathways and inflammatory cytokines such as IL-17 (Majumder et al., 2019). On the other hand, the same type of cells can also help induce tolerance within the T cell population. TRC have the ability to present peripheral tissue antigens within the T cell zone (Fletcher et al., 2010). These cells also produce nitric oxide (NO) which helps inhibit the proliferation of activated T cells (Siegert et al., 2011). These processes can help attenuate self-reactivity.

A subset of fibroblasts can also be found within the medullary cords which help define and maintain the plasma cell niche (Huang et al., 2018). Similar to other fibroblast cell populations, these cells create a reticular cell network within the medullary cords, but they are functionally distinct from other fibroblastic populations in the T and B cell regions of the lymph node. The medullary FRC express soluble proteins such as CXCL12, CCL2, IL-6, and APRIL which fosters the migration, localization, survival, and function of plasma cells (Huang et al., 2018) (Figure 1J).

Importantly, it should be emphasized that cells from the mesenchymal lineage often interact with cells from the endothelial lineage indicating crosstalk between stromal cell populations. Pericytes and BEC are one example of this interaction. Pericytes surround small blood vessels and are essential for vessel maturation and stabilization (Dore-Duffy and Cleary, 2011; Navarro et al., 2016). Angiogenesis begins with the detachment of pericytes from endothelial cells and ends with the stabilization of the new vessel through the recruitment of new pericytes (Jain, 2003). New functional roles of pericytes have also been discovered outside of their structural role. These functions include involvement in immune cell trafficking through the expression of adhesion molecules such as ICAM-1 and VCAM-1, or contributing to the onset of innate immune responses through the expression of various cytokines and chemokines. Alternatively, they may induce T cell anergy through their expression of PD-L1 (Maier and Pober, 2011; Guijarro-Munoz et al., 2014; Navarro et al., 2016) (Figure 1G). Overall, similar to the endothelial-derived cell populations, the mesenchymal-derived cell populations are diverse and influence many different aspects of the immune responses within the lymph node.

### Stromal Cells in Healthy Homeostasis of Non-lymphoid Tissues

The stromal cell populations identified in lymph nodes are also found throughout non-lymphoid tissues. In this setting, stromal cells not only provide a structural and nutritional support for parenchymal tissues, but also help mold the immune profile within various tissues by shaping the transcriptional program of the hematopoietic compartment within each organ. Many of these functions are highly organ specific and help to create an optimal microenvironment for the function of the organ (Krausgruber et al., 2020).

Mesenchymal-derived stromal cells help to shape the organ microenvironment through the production of retinoic acid which regulates innate immune cells in the mucosal lining. For example, retinoic acid production by Wilm’s Tumor 1 (WT1) mesothelial and fibroblastic stromal cells helps to maintain both homeostasis and the GATA6 transcriptional program in cavity resident macrophages (Buechler et al., 2019). Additionally, retinoic acid production by stromal cells in the intestinal lamina propria promoted a two-way “crosstalk” between stromal cells and mucosal dendritic cells (Vicente-Suarez et al., 2015). Mesenchymal stromal cells can also promote adaptive immune regulation. In the lung, mesenchymal subsets produce IL-33 which creates an immune suppressed environment. This environment supports ILC2s and regulatory T cell (T_reg_) differentiation and is crucial for proper function of the lung (Dahlgren et al., 2019).

Mesenchymal stromal cells are also important in immune cell differentiation as well as tissue health within the liver environment. Hepatic stellate cells, a type of liver-specific stromal cell (pericytes), produce CSF-1, IL-34, and bone morphogenetic proteins which play a role in the differentiation of monocytes into Kupffer cells (Bonnardel et al., 2019). Kupffer cells act as tissue resident macrophages in the liver. These cells are essential in maintaining proper liver function and act as the first line of defense against bacterial infections (Naito et al., 1997).

Most types of non-lymphoid tissues, especially the skin dermis and various mucosal membranes are drained through an extensive network of lymphatic vasculature (Petrova and Koh, 2018). The lymphatic vasculature is lined with heterogeneous populations of LEC with functions specific to their location, with a complexity perhaps best illustrated in the small intestine. Not only do the LEC and other stromal cells in the small intestine vasculature function to transport intestinal fluid and lymph, but they are also involved in the uptake and transport of dietary fats (Bernier-Latmani and Petrova, 2017). In mammals, dietary lipids are packaged as chylomicrons, or large triglyceride loaded particles. These particles can transport more than just triglycerides. Fat soluble vitamins and drugs, along with microbiota products such as LPS can also be transported in this way. Intestinal lymph is then transported to the mesenteric lymph nodes where molecules can be surveyed by the immune system or released into blood circulation (Bernier-Latmani and Petrova, 2017). Importantly, this process allows for lipid soluble drugs to bypass the liver, where they will most likely be degraded, and access their target organ (Petrova and Koh, 2018).

Intestinal lymphatic vessels are also important in the maintenance of tolerance to commensal bacteria and food antigens. CCR7^+^ dendritic cells migrate into the intestinal lymphatic system via the CCL21 gradient generated by intestinal LEC. Trafficking of these dendritic cells to the mesenteric lymph node help generate T_reg_ cells and induce tolerance (Forster et al., 2008; Pabst and Mowat, 2012). Presentation of LEC archived antigens to CD103^+^ dendritic cells allows for the transport of food or pathogen antigens to the mesenteric lymph node (Tamburini et al., 2014) and thus plays a role in the induction of oral tolerance or mucosal immunity.

In summary, a large body of literature evidence gathered in recent studies demonstrates that stromal cell–immune interactions are not only necessary for the proper development and function of immune cells within the lymphoid organs, but also play an essential role in supporting and regulating immunity and tolerance in non-lymphoid target tissues in health.

## Stromal Cells in Chronic Inflammatory States of Autoimmunity and Malignancies

Inflammation is an essential defense mechanism to restore tissue homeostasis after injury. It is typically resolved by well-controlled immune responses and the participation of organ protective and/or antimicrobial mediators such as resolvins, protectins, and maresins (Serhan and Levy, 2018). If the initial trigger persists and/or immune dysregulation occurs, chronic inflammation can occur (Nathan and Ding, 2010). This scenario is well-illustrated in autoimmune diseases and cancer, two of the major disease burdens in human populations. Chronic inflammation underlies and perpetuates autoimmune diseases and is also believed to play a major role in the development of cancer (Multhoff et al., 2011; Maria et al., 2018). This process has long been known to involve a variety of cellular and molecular mediators in the immune compartment. With the results from more recent studies, it is now well-recognized that stromal cell interactions with the immune system can contribute to the development and maintenance of the chronically inflamed state (Buckley et al., 2015).

### Stromal Cells in Autoimmune Diseases: RA and IBD as Examples

The initial recognition for a key role of stromal cells in autoimmune diseases came from the finding that a subset of thymic stromal cells, thymic medullary epithelial cells, shape central immune tolerance induction by self-antigen presentation (Anderson et al., 2002). Since then, studies by many groups have established a key role for LNSC as well as stromal cells in target tissues in a number of autoimmune disorders (Pistoia and Raffaghello, 2017; Ramaglia et al., 2021; Royer and Cook, 2021; Sim et al., 2021). In this review, we survey the role of stromal cells in non-lymphoid target tissues identified in two autoimmune diseases, rheumatoid arthritis (RA) and inflammatory bowel disease (IBD) because of the large pool of evidence gathered regarding these two diseases.

**FIGURE 2:**
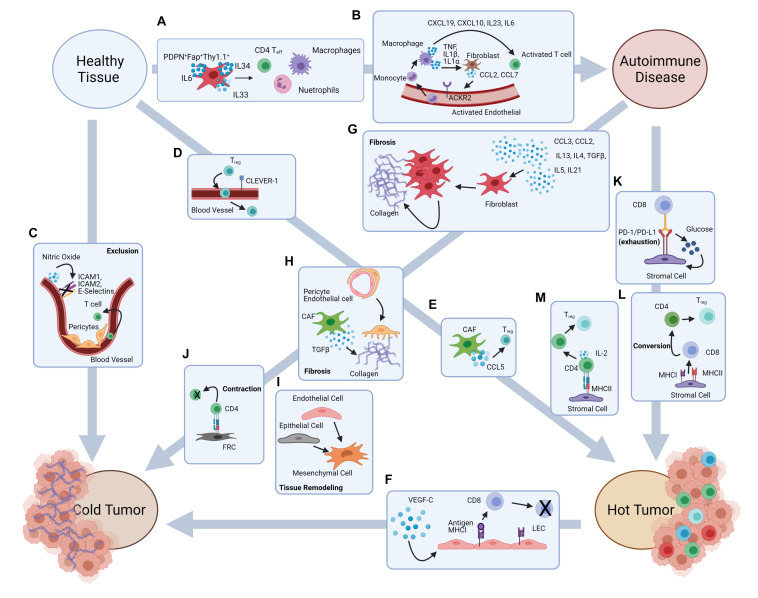
Stromal cell-mediated potential mechanisms underlying the putative pathways from autoimmune damage to cancer development. Autoimmune disease onset and cancer development are both characterized by inflammation and immune dysregulation. Literature evidence suggests some putative pathways that may associate autoimmunity and cancer development, and possibly explains the autoimmune etiology in some types of cancer. **(A)** Fibroblasts contribute to the recruitment of inflammatory cells in autoimmune disorders such as RA. **(B)** Activated fibroblasts and endothelial cells contribute to inflammation within the large intestine. **(C)** Abnormal BEC structure and pericyte interactions lead to T cell exclusion in the TME, while NO production downregulates the expression of adhesion markers on blood vasculature. **(D)** CLEVER-1 expression by blood vessels leads to T_reg_ recruitment into the TME. **(E)** CCL5 production by CAF contribute to T_reg_ recruitment into the TME. **(F)** VEGF-C production increases LEC proliferation and antigen presentation leading to CD8 T cell apoptosis. **(G)** Inflammatory signals lead to fibroblast proliferation and collagen production causing fibrosis. **(H)** Pericyte transition into fibroblast like cells and TGFβ expression by CAF contributes to fibrosis. **(I)** Endothelial cell to mesenchymal cell transition and epithelial to mesenchymal cell transition. **(J)** Antigen presentation by FRC leads to CD4 T cell contraction. **(K)** PD-1/PD-L1 interactions leads to metabolic reprograming in both CD8 T cells and stromal cells. **(L)** MHCI and MHCII expression by stromal cells contributes to CD8 to CD4 lineage conversion. **(M)** Antigen presentation to CD4 T cells by stromal cells leads to T_reg_ differentiation.

Rheumatoid arthritis is an autoimmune disease mainly affecting the body’s joints. Unresolved inflammation leads to compromised joint function (Gay et al., 1993), and in severe cases may affect internal organs (Bullock et al., 2018). Synovial fibroblasts, which are derived from the mesenchymal lineage of cells, mediates the inflammation within the joints (Pap et al., 2000). Single cell transcriptomics have found that this population of fibroblasts is not homogenous. The heterogeneity of this population could be very relevant in the discovery of new therapeutic targets, especially in terms of personalized medicine. In preclinical models, cell transfer studies in mice have shown that adoptive transfer of one subset of fibroblast leads to a pathology similar to RA while adoptive transfer of a second fibroblast population leads to a pathology similar to osteoarthritis which is characterized mainly by cartilage damage and not inflammation (Croft et al., 2019). The RA associated fibroblast population was characterized as PDPN^+^Fap^+^THY1.1^+^ and found to produce cytokines such as IL-6, IL-34, and IL-33. These cytokines are associated with the recruitment of pathogenic CD4 T cells, macrophages, and neutrophils to the synovial space ultimately contributing to inflammation of the joint (Figure 2A). Treatment for RA is complicated, mainly due to the diversity of disease pathology within the patient population. Identification of the stromal cell subsets in the inflamed tissues and better understanding of their role in the microenvironment could aid in the development of new treatments for RA.

The subsets of stromal cells involved in chronic inflammation may vary in different disease settings. In IBD, single cell analysis has found a distinct inflammatory population of mesenchymal stromal cells within the intestine of both mice and humans affected by colitis (Martin et al., 2019). This proinflammatory population was enriched for inflammatory, fibrotic, and cancer associated genes (Martin et al., 2019). In other studies, the LEC population has been shown to plays a role in IBD pathology. In experimental models of colitis, impaired intestinal lymphatic vascular function exacerbates intestinal inflammation and worsens symptoms associated with colitis. D6 decoy chemokine receptor deficiency in mice also causes increased severity of colitis symptoms. This molecule is highly expressed by LEC and is necessary to restrict irregular inflammatory leukocyte adhesion to lymphatic vessels (McKimmie et al., 2013).

Collaboration between stromal cell populations can also exacerbate autoimmune pathology. A single cell analysis of Chron’s Disease legions showed signaling between fibroblast and endothelial cells contribute to inflammation within the large intestine lamina propria. The activated fibroblast population produces CCL2 and CCL7 which interacts with ACKR2 on activated endothelial cells. This leads to recruitment of circulating monocytes which differentiate into macrophages. These inflammatory macrophages produce cytokines such as TNF, IL-1β, and IL-1α which further activate the fibroblast population while also recruiting pathogenic CD4 T cells into the large intestine (Martin et al., 2019) (Figure 2B). Currently treatments for IBD typically target the immune system to dampen chronic inflammation (Axelrad et al., 2016). The efficacy of current interventions can substantially improve if effective approaches can be developed to target the stromal compartment. For that purpose, it is important to understand how the stromal cell compartment and which subset(s) play a major role in perpetuating chronic inflammation.

Taken together evidence from a number of autoimmune disorders, it is now recognized that autoimmune diseases are a major class of inflammatory diseases whose pathology may be partially caused and exacerbated by the interactions of stromal cells and immune cells. Further investigation into these interactions may increase understanding of the onset and pathology of these heterogeneous diseases, and eventually lead to new avenues for therapeutic development.

### Stromal Cells in Cancer

The role of stromal cells has been generally recognized in the development of cancer; in particular, stromal cells are now well known to have a critical role in both the formation and function of the tumor microenvironment (TME). Within the TME, stromal cells can play an important role in modulating immune cells. TME stromal cells are not derived from a malignant cell lineage; therefore theoretically these cells should function similarly to their counterparts in healthy tissues (Turley et al., 2015). However, recent studies have shown that this is not the case. Although the stromal cells within the TME may not be malignant, their function tends to be altered within the tumor tissues. These alterations can affect the function of lymphocytes within the TME and influence tumor growth, metastasis, and chemoresistance (Turley et al., 2015). It can also be theorized that stromal cell alterations may contribute to the development of either “hot” or “cold” tumors, or potentially the conversion between the two.

The BEC subset plays an important role in the migration of immune cells into tissues. Within the tumor, it has been shown that trafficking of antigen specific T cells is dysfunctional. Some of this immune trafficking dysfunction can be contributed to abnormal structure of vasculature within the tumor (Griffioen et al., 1996a). Tumor BEC are irregularly shaped and have loose intercellular connections while pericytes are also abnormal within tumor vessels. Pericytes loosely associate with the BEC and the basement membrane which contributes to the leakiness of tumor vasculature (Castermans and Griffioen, 2007). The effects of the abnormal structure of tumor vasculature are exacerbated by a change in the adhesive properties of these cells. It has been shown that BEC within the tumor vasculature have reduced expression of E-selectin. This reduction leads to impaired lymphocyte recruitment. Tumor BEC also poorly express ICAM-1, ICAM-2, and VCAM-1 (Griffioen et al., 1996a). These molecules are involved in lymphocyte extravasation from circulation into tissues. Changes in NO production by vasculature stroma can also affect lymphocyte adhesion and extravasation. NO production helps maintain the vasculature in a resting state. During homeostatic conditions, production of NO by BEC inhibits lymphocyte tissue infiltration (Griffioen et al., 1996b). Within the TME several factors can alter the production of NO. These factors include VEGF-A and fibroblast growth factor 2 (FGF2) which stimulate NO synthases leading to the catalysis of NO production by BEC. This process leads to the inhibition of endothelium activation and reduced expression of adhesion molecules (Bouzin et al., 2007) (Figure 2C).

Evidence has also been found to suggest tumor endothelium favors the extravasation of immunosuppressive lymphocyte populations such as T_reg_ cells. Of note, T_reg_ infiltration has been associated with decreased survival in many types of cancer. One example is the increased expression of CLEVER-1 in the BEC population found in human hepatocellular carcinoma. CLEVER-1 was found to preferentially mediate the infiltration of T_reg_ populations into the tumor (Shetty et al., 2011) (Figure 2D). Cancer associated fibroblasts (CAF) also play a role in T_reg_ recruitment. CAF are defined as being FAP^+^αSMA^+^ and are the most abundant stromal cell population within the tumor microenvironment (Kalluri and Zeisberg, 2006). In mammary carcinoma increased CCL5 production by CAF is associated with increased T_reg_ recruitment, which may in turn facilitate the growth of tumors by effecting the anti-tumor immune response within the TME (Tan et al., 2011) (Figure 2E).

Modulation of immune cell recruitment to the TME is not the only way stromal cells can impinge on the immune profile. Stromal cells can alter tolerance induction and immune cell differentiation within the TME. LEC in melanoma have the ability to directly modulate the adaptive immune response within the tumor microenvironment. A study by Lund et al. (2012) found when the LEC population was genetically engineered to express VEGF-C, a lymphangiogenic factor which correlates with metastasis and poor prognosis, the antitumor immunity response was hindered. This hindrance was due to the cross-presentation of tumor antigens by the LEC population ultimately leading to the apoptosis of antigen specific CD8^+^ T cells and tolerance induction to tumor antigens (Lund et al., 2012) (Figure 2F). Deletion of lymphocyte populations within the TME may be one potential mechanism leading to the conversion of a “hot” tumor, which contains immune cells in the TME, to its “cold” counterpart which is characterized as being void of immune cells (Duan et al., 2020).

## Stromal–Immune Interaction Underpinning the Pathways From Autoimmunity, Inflammation to Cancer

While autoimmunity may function as a “double agent” in cancer (Multhoff et al., 2011; Toomer and Chen, 2014; Maria et al., 2018) and its anti-tumor effect may indeed involve stromal damages or alteration, this review explores potential mechanisms of altered stromal–immune interaction in autoimmune inflammation that leads to tumorigenesis. We focus on a few of the many potential new mechanisms, keeping in mind the limitation of our scope of knowledge, on how stromal cell pathobiology may underline the immunological and metabolic pathways from autoimmunity, inflammation to cancer.

### Stromal Pathology Shared in Autoimmunity and Cancer: Fibrosis and Tissue Remodeling

At the histopathological level, among the most conspicuous alterations shared by autoimmune diseases and cancer are tissue fibrosis and tissue remodeling. Chronic tissue damage by autoimmune inflammation leads to fibrosis and tissue remodeling. Fibrosis and tissue remolding are also hallmarks of malignance pathology, but whether and how the fibrosis and tissue remodeling initiated by autoimmune damage might contribute to cancer development is still unknown.

Fibrosis is characterized as the deposition of collagen and other extracellular matrix components leading to a buildup of fibrous connective tissues. During chronic inflammatory damage, the stromal cell compartment adapts to the fibrotic development to fortify the tissue barrier and prevent catastrophic breaches, a process that also leads to remodeling of the parenchymal tissues, and potential disrupted organ function (Wynn, 2007; Zhang and Zhang, 2020). It has also been shown to contribute to tumor development, but the exact mechanisms remain largely unknown (Yamauchi et al., 2018). Nevertheless, the immunological consequences of chronic fibrosis can be speculated, such as lost immunogenicity besides the physical impediment of immune–tumor interactions. It is known that effective antitumor immunity can be elicited by targeting either self-antigens or tumor-specific antigens, the latter of which is rare among the abundance of the former. So how could the tumor cell lineage develop in the setting of potent immune cells against the specific self-antigens? Could it be possible that tissue remolding and the development of fibrotic tissues in the autoimmune setting leads to the down regulation of these specific autoantigens in turn allowing tumor cells to escape detection? In other words, could the chronic autoimmune destruction “edit” out the cells expressing the target antigens and eventual lineage escape due to their lack of self-antigens recognized by the specific autoimmune T cells?

Inflammation and tissue damage repair are essential mechanisms of homeostatic maintenance. During acute inflammatory events tissue damage is repaired in a controlled and systematic manner. However, during chronic inflammation these mechanisms are often disrupted. For instance, fibrotic tissue development often occurs in patients suffering from IBD, an autoimmune disease which also has links to cancer development (Rieder and Fiocchi, 2009). Moderate fibrosis is a normal occurrence in a majority of patients suffering from IBD. In these cases, the fibrosis does not show any clinical manifestations, but approximately 30% of cases experience severe fibrosis (Silverstein et al., 1999). It has traditionally been believed that fibrosis is caused by chronic exposure to inflammation which cause fibroblasts to proliferate and deposit collagen leading to the development of scar tissues over an extended period of time (Figure 2G). This view of fibrosis often simplifies the complicated process which involves not just fibroblasts, but also other stromal cells along with many different immune cells, cytokines, and chemokines (Rieder and Fiocchi, 2009). For example, pericytes have been found to contribute to fibrosis, especially in the skin. Pericytes have the ability to differentiate into fibroblast like cells as a response to inflammation. During these events pericytes detach from endothelial cells, migrate into the surrounding tissues and produce collagen ultimately contributing to fibrosis in these tissues (Sundberg et al., 1996) (Figure 2H).

As mentioned in earlier sections stromal cells are derived from either the mesenchymal or the endothelial cell lineages. In the case of fibrosis, transformations of stromal cell lineages can occur even though stromal cells in tumors may not necessarily originate from the malignant lineage. During the transformation fibroblasts are generated from cells which did not originate from the mesenchymal origin. The transformation processes include epithelial-to-mesenchymal transition and endothelial-to-mesenchymal transition (Frid et al., 2002; Kalluri and Neilson, 2003) (Figure 2I). Many of the mediators needed for these transitions are present in the autoimmune setting. Additionally, the populations of cells undergoing transformations are extremely heterogeneous with various functions.

Investigation into which subsets of stromal cells may undergo transformation could help to develop new therapies with more specific targets. These cell lineage transformations can also contribute to tumor malignance, especially in light of their interaction with immune cells, although these interactions can often be complicated and vary in different disease settings (Cho et al., 2018; Choi et al., 2020). For instance, T_reg_ have been shown to be pro-fibrotic in some disease states, and anti-fibrotic in other disease states (Zhang and Zhang, 2020). Metabolites, cytokines or growth factors from parenchymal or inflammatory cells may also influence the lineage fate of stromal cells. Therefore, it is important to understand these complex relationships and interactions between immune cells, their byproducts, and stromal cells in order to better understand how the process of fibrosis development and tissue remolding may underlie the development of cancer in the autoimmune setting.

### Potential Effect of Inflammatory Stromal Responses on T Cell Exhaustion, Lineage Conversion and Exclusion From Autoimmunity to Cancer

Immune dysregulation of T cells plays a key role in initiation, execution and perpetuation of autoimmune tissue damage. Abnormal or ineffective T cell response is also a key feature of cancer and a target of immune therapy. Given the alteration of stromal cell contexture by inflammation, fibrosis and tissue remodeling in both autoimmune diseases and cancer, it is conceivable that T cell regulation and/or dysfunction impinged by the stromal–immune interaction in autoimmune settings are projected to malignant tissues, not only likely in “hot” tumors that have abundant yet ineffective *in situ* immune reaction, but also possibly in “cold” tumors that are largely devoid of inflammatory infiltrates (Figure 2).

Autoimmune T cells can drive inflammatory tissue damage and initiate cancer development (Nguyen et al., 2013; Miska et al., 2018). The exact state of the T cell differentiation in the autoimmune and tumorigenic conditions remains to be characterized. Recent studies have brought attention to a state of T cell differentiation called exhaustion in chronic inflammatory settings (Wherry, 2011). And indeed, T cell exhaustion may be a new potential mechanism that helps connect the “dots” between autoimmunity and cancer. T cell exhaustion is commonly seen during chronic infection and in the TME. Exhaustion occurs when T cells are exposed to persistent antigen and or inflammatory signals. T cell exhaustion can be seen in both the CD8 and CD4 T cell lineages and is marked by both phenotypic and mechanistic difference when compared to other dysfunctional T cell states. The exhausted T cells have reduced effector function, memory properties, and increased expression of inhibitory receptors (Wherry, 2011). Additionally, T cell exhaustion can also be seen in the autoimmune setting but is more often viewed positively (McKinney et al., 2015). In this regard, stromal cells could potentially drive the switch to the exhausted state in T cells as a mechanism to control the chronic inflammation. Then, might this mechanism promote the development of cancer within the tissue in the process of progression from chronic inflammation to tumorigenesis?

For many types of cancer in their fully developed states, it is well-recognized that T cell exhaustion is prevalent and needs to be overcome for the disruption of TME (Jiang et al., 2015; Hashimoto et al., 2018; McLane et al., 2019). Many immunotherapies target co-inhibitory pathways as a way to overcome T cell exhaustion and promote immunity against cancer or chronic infection (Jiang et al., 2015; Hashimoto et al., 2018; McLane et al., 2019). On the other hand, in the case of autoimmune diseases, exhaustion may improve patient outcome. Untreated patients suffering from an active autoimmune disease who present a transcriptional signature similar to that seen in exhausted CD8 T cells have a more favorable long-term prognosis. This was seen in antibody associated vasculitis (AAV), IBD, and systemic lupus erythematosus (SLE) (McKinney et al., 2015). Therefore, in the autoimmune diseases, inducing T cell exhaustion could potentially reduce relapse and increase favorable prognosis within patients suffering from active diseases. Interestingly, those exhausted cells may maintain some of their effector function, suggesting that these T cells may not be permanently exhausted or may have the ability to switch between the exhausted and effector states. This could account for the cycle of relapse which is observed in patients suffering from autoimmunity. The role of stromal cells in the induction of T cell exhaustion during autoimmunity has not been well studied. However, extrapolating from of the functions of stromal cells in the TME it is reasonable to hypothesize that stromal cell interactions with immune cells could play a role in the induction and maintenance of the exhausted T cell state.

While the molecular nature of T cell exhaustion in autoimmunity remains to be elucidated, T cell exhaustion as a hallmark of the TME is well characterized in a number of cellular and molecular aspects, which is believed to be induced by several different mechanisms. The best characterized mechanism is perhaps the PD-1/PD-L1 pathway (Jiang et al., 2015; Hashimoto et al., 2018; McLane et al., 2019). It is well known that the level of expression of PD-L1 on tumor cells correlates with patient prognosis while anti-PD1 immunotherapy has been shown to be successful in many cases (Jiang et al., 2015; Hashimoto et al., 2018; McLane et al., 2019). Less known, and strikingly, is the fact that PD-L1 is abundantly expressed on stromal cells and is often upregulated within the TME, despite the non-malignant origin of the stromal cells (Schworer et al., 2019). PD-1/PD-L1 interactions have been shown to alter glucose metabolism in both the stromal cell and the T cell subsets, driving T cells toward the exhausted state. PD-1/PD-L1 interactions enhance glucose uptake of tumor stromal cells while simultaneously decreasing glucose uptake of T cells. This metabolic reprograming favors an immunosuppressed TME (Schworer et al., 2019) (Figure 2K).

The distinct profiles of cytokine and chemokine expression by stromal cells induced under inflammatory conditions could impact T cell differentiation much beyond a state of an active effector versus exhaustion. Of note, perturbation of cytokine signals can as profound an effect on T cell lineage specification even in thymic development of CD8^+^ and CD4^+^ T cells (Etzensperger et al., 2017), the two main lineages of T cells driven by MHC class I (MHCI) and MHC class II (MHCII), respectively, selection (Doyle and Strominger, 1987; Norment et al., 1988; Veillette et al., 1988), although it has long been thought that lineage commitment, once completed in the thymus, is stable due to unique transcriptional programing (He et al., 2005; Sato et al., 2005; Hedrick, 2012). Indeed, CD4 and CD8 T cell lineage plasticity manifest in some peripheral microenvironments (Mucida et al., 2013; Reis et al., 2013; Lui et al., 2015). Of particular relevance to a potential new mechanism of immune regulation, CD8^+^ T cells have been shown to convert to the CD4^+^ T cell lineage including immune suppressive Foxp3^+^ regulatory T (T_reg_) cells (Lui et al., 2015).

The mechanisms underlying these lineage conversions are still mostly unknown, but there is suggestive evidence for a role of MHC expression in stromal cells. It has been shown that MHCII expression is required for the conversion of CD8^+^ T cells to CD4^+^ T_reg_ cells in the periphery (Lui et al., 2015). As discussed in previous sections, stromal cells have been found to express MHCII both intrinsically and acquire MHCII extrinsically. It has also been shown that MHCII expression by stromal cells can induce the T_reg_ phenotype in conventional CD4^+^ T cells, therefore indicating a potential role for stromal cells to directly influence T cell lineage commitment in the periphery (Nadafi et al., 2020) (Figures 2L,M). Antigen presentation by stromal cells has also been shown to lead to CD4 T cell contraction. In a study published by Abe et al. (2014), it was shown that during viral infection antigen presentation by LNSC, specifically FRC, leads to T cell contraction (Figure 2J). This mechanism may be a potential way to prevent continued CD4 effector T cell response once antigen has been cleared. It is theorized that this mechanism may also play a role in non-lymphoid tissues (Abe et al., 2014), and therefore could be an important mechanism in the control of autoreactive T cells. Potentially in the autoimmune setting, antigen presentation by fibroblast populations could help to eliminate pathogenic CD4 T cells, similar to what is seen in the lymph node during infection. Alternatively, this mechanism could also underlie the development of cancer from autoimmunity. Deletion of autoreactive T cells in the autoimmune setting could cause malignant cells to go undetected, promoting the development of cancer. Likewise, stromal cells have also been shown to play a role in immune cell differentiation and recruitment in both the autoimmune and cancer settings (Turley et al., 2015; Petrova and Koh, 2018; Koliaraki et al., 2020). Exploration into these mechanisms could also help better understand the connection between autoimmunity and cancer. For instance, how does the inflammatory environment affect lineage conversion and immune cell differentiation, and how might these changes affect cell function? The answers to these questions may lead to novel therapies for both autoimmune diseases and cancer, especially given with their often shared inflammatory states sustained by immune cells or cytokines.

Of note, in the tumor microenvironment, the presence of the various immune constituents, regardless of effectiveness, characterizes the tumor as “hot” and in general a more likely target responding to immune therapy, unlike its “cold” counterpart that is largely devoid of immune cell infiltrates. How a tumor microenvironment evolves to be immune “cold” remains essentially unknown (Duan et al., 2020)? It is possible that “cold” tumors may not necessarily begin as “cold,” especially if it has a potential autoimmune origin. Rather, an active process of immune exclusion may have occurred from autoimmunity to cancer which is possibly contributed by altered stromal cells. In this regard, it is worth noting that in autoimmune arthritis, expression of FAP and PDPN marks a subset of synovial fibroblasts that promote autoimmune inflammatory damage (Croft et al., 2019), while FAP^+^ PDPN^+^ fibroblasts in the cancer setting may mediate the exclusion of T cells from tumor infiltration, through TGFβ signaling and fibrosis as well as nitric oxide-mediated T cell suppression (Monteran and Erez, 2019). Overall, the evidence from autoimmunity and cancer studies, taken together, suggest that stromal cells altered by chronic autoimmune inflammation may play a critical role in underlining the pathways to cancer development in some cases, regardless of the eventual outcome of “hot” or “cold” tumors.

## Conclusion and Future Directions

Both autoimmune diseases and cancer are heterogeneous diseases which vary greatly from patient to patient. With this heterogeneity comes difficulty in identifying common causative mechanisms and universal treatments for either category of the diseases. Nevertheless, recent studies suggest that there may be a number of mechanisms linking these two complicated diseases together. One of the major advances is the discovery of new roles for stromal cells in immunological and metabolic regulation that are far beyond their traditionally assigned functions in providing structural and nutritional support. Thus, stromal cells could present attractive therapeutic targets against both autoimmunity and cancer, with immune therapy as well as metabolic interventions. A better understanding of stromal cell intrinsic and extrinsic regulation of immune cells, such as T cell exhaustion, contraction or lineage conversion, will likely lead to a plethora of novel therapeutic approaches. New discoveries in metabolic reprograming of both stromal and immune compartments will also likely lead to novel and exciting venues for preventions and treatments of autoimmune disorders and cancer. Those approaches could substantially help curtail the rising incidences of both autoimmunity and cancer, especially among the young, and thus reduce the burden of these two major types of diseases on humanity.

## Author Contributions

AH researched and wrote the manuscript. AH and ZC designed the figures and conceived the theme and framework. ZC reviewed and edited the manuscript. Both authors contributed to the article and approved the submitted version.

## Conflict of Interest

The authors declare that the research was conducted in the absence of any commercial or financial relationships that could be construed as a potential conflict of interest.
